# Controlling the hydration of the skin though the application of occluding barrier creams

**DOI:** 10.1098/rsif.2012.0788

**Published:** 2013-03-06

**Authors:** Emma Sparr, Danielle Millecamps, Muriel Isoir, Véronique Burnier, Åsa Larsson, Bernard Cabane

**Affiliations:** 1 Physical Chemistry, Lund University, PO Box 124, 22100 Lund, Sweden; 2 L'ORÉAL, 188 rue Paul Hochart, 94550 Chevilly Larue Cedex, France; 3 PMMH, ESPCI, 10 rue Vauquelin, 75231 Paris cedex 05, France

**Keywords:** responding membrane, water transport, hydration, permeability, model emulsions

## Abstract

The skin is a barrier membrane that separates environments with profoundly different water contents. The barrier properties are assured by the outer layer of the skin, the stratum corneum (SC), which controls the transepidermal water loss. The SC acts as a responding membrane, since its hydration and permeability vary with the boundary condition, which is the activity of water at the outer surface of the skin. We show how this boundary condition can be changed by the application of a barrier cream that makes a film with a high resistance to the transport of water. We present a quantitative model that predicts hydration and water transport in SC that is covered by such a film. We also develop an experimental method for measuring the specific resistance to water transport of films made of occluding barrier creams. Finally, we combine the theoretical model with the measured properties of the barrier creams to predict how a film of cream changes the activity of water at the outer surface of the SC. Using the known variations of SC permeability and hydration with the water activity in its environment (i.e. the relative humidity), we can thus predict how a film of barrier cream changes SC hydration.

## 1. Introduction

**Figure 1.**
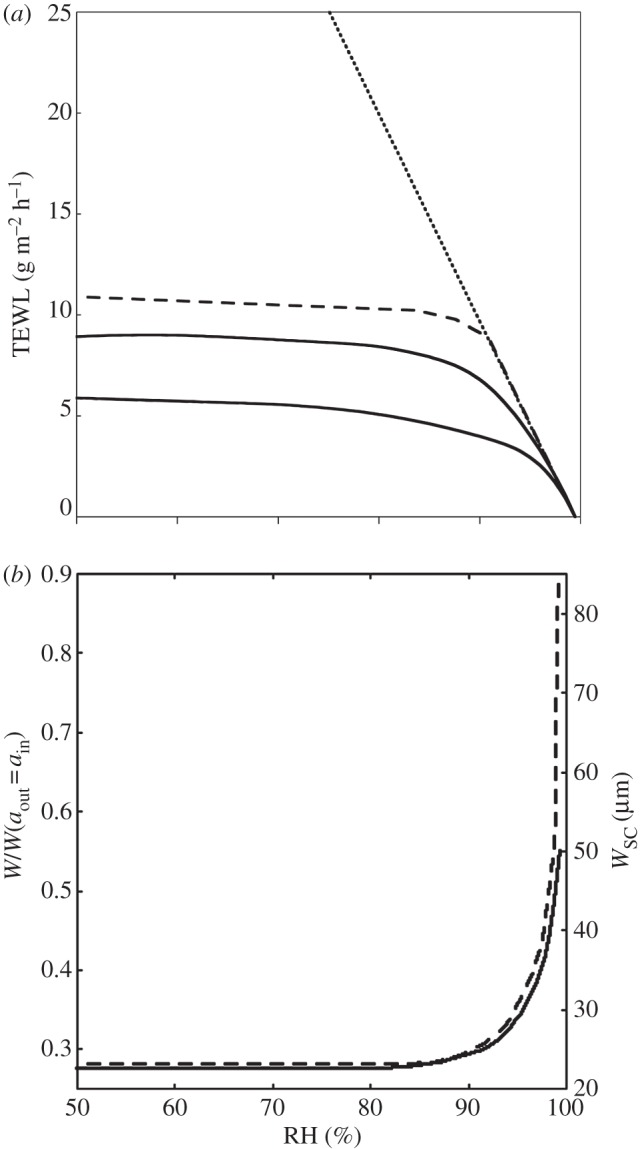
(*a*) The influence of RH on the water flux (TEWL) and (*b*) the thickness for SC and a model membrane composed of stacked bilayers where one side of the membranes faces a physiological solution (correspond to 99.6%RH), and the other side faces an environment that is defined by its RH. Solid lines, fit to experimental data obtained for intact SC [2]; dashed line, calculated data for a responding membrane [3]; dotted line, calculated data for a non-responding membrane [4]. In figure (*b*), the calculated data are presented as the change in thickness relative to the fully swollen membrane (*a*_out_ = *a*_in_) (left axis). The experimental data for SC are given as measured thickness (right axis).

The barrier membrane in the human skin has a major, vital function to limit water evaporation from the body, and to prevent the entrance of exogenous chemicals. Yet, there is a transepidermal water loss (TEWL) of about 100–150 ml per day and square metre of skin surface through healthy skin [1] (figure 1). The driving force for the TEWL is the large difference in water activity between the water-rich tissue inside the body and the very dry environment outside the body (characterized by the relative humidity (RH), in air). As a comparison, the physiological conditions inside the body correspond to *ca* 99.6%RH, and in-house external environment is generally between 40%RH and 60%RH. This implies a rather extreme gradient in water activity across the skin membrane [5,6]. The magnitude of the TEWL depends on the magnitude of this water gradient, and on the water permeability in the skin barrier membrane.

Healthy skin is unusual as a membrane in that its permeability changes with water activity (or RH) outside the body, i.e. it is a responding membrane [7–9]. The skin barrier function is assured by the very outer epidermis layer, the stratum corneum (SC) [10]. The hydration of the SC membrane is crucial in regulating the barrier properties [2,7], and it is also a determinant factor to other vital functions of healthy skin in relation to mechanical properties, appearance and the enzymatic activity in SC [11,12]. Under normal conditions, the water supply for skin hydration and TEWL is from within the body. At steady state, the SC is hydrated at a level that is determined by the water gradient across the skin, and this hydration determines its permeability [2,7,13]. In healthy skin, the relation between hydration and permeability produces a TEWL that remains nearly constant over a wide range of ambient RH [7,14,15].

Since the SC hydration is determined by water activity at its outer surface (*a*_out_), it can be changed by the application of a cosmetic or pharmaceutical occluding film that separates the SC from the outside environment. The film can be described in terms of its specific resistance to diffusive transport of water (*ρ*). A film with high *ρ* will increase *a*_out_ above its value in the external environment, *a*_RH_. Since the SC permeability changes with *a*_out_, it is not *a priori* obvious what the effects on TEWL and SC hydration will be. Skin occlusion can also lead to enhanced percutaneous uptake of topically applied compounds, e.g. drugs [8,16], and it can influence skin biology, skin pH and wound healing [17–20].

We present a theoretical model that couples transport and hydration in responding skin membranes after the application of a film on the skin. We also present an experimental method to determine the specific resistance to water transport of films made of barrier creams. In this work, we assume that the components of the cream (other than water) do not penetrate into the SC. Indeed, penetration of small molecules used as ‘moisturizers’, such as glycerol and urea, also influences skin hydration through a mechanism that is completely different from what is described here [21–24]. With the assumption of no penetration of foreign molecules, we can use the properties of the intact SC and the measured properties of the barrier creams to make a quantitative prediction of the behaviour of the skin + cream composite. Thus, we obtain a quantitative tool that predicts how a film of barrier cream affects the water activity in the upper SC, its hydration and the TEWL.

## 2. Theory

### 2.1. A self-consistent model for the stratum corneum

Numerous studies have shown that the barrier properties and hydration of SC depend on the external RH [7,8,13–16,25–28]. The SC is thus a responding membrane, which cannot be described with a simple permeability constant [9,29]. We have previously constructed a self-consistent model that describes transport in responding membranes [3,29], and this forms the basis for the model presented in this paper. The theoretical analysis was previously performed for model membranes composed of stacked lipid bilayers, as a mimic of the extracellular lipids in SC [4]. The model predicts the water flux (TEWL) and the water content gradient (∂*W*/∂*z*) across the membrane as functions of the boundary conditions, which are expressed by the chemical potential of water (or the water activity) on either side of the membrane. On the internal side, the boundary condition is always *a*_in_ = 0.996 (corresponds to 99.6% RH). On the external side, it varies with the environment, and in particular with the RH of the ambient air. In practice, we can assume that *a*_out_ = RH (see §3). The theoretical analysis takes into account that the gradient in water activity can lead to heterogeneous swelling and phase transformations within the membrane, which in turn affects the molecular environments and thus the local diffusion properties. One outcome of these studies is that a gradient in water activity across a responding lipid membrane can be used as a switch to regulate the membrane properties [29].

Comparisons between the predictions from the theoretical modelling [3,29] and experimental data obtained for intact SC are shown in figure 1 (water flux and SC thickness as function of RH [7]). The comparisons demonstrate that the model for responding membranes captures the essential behaviours of the human skin. Indeed, figure 1*a* shows that the flux remains approximately constant at all RH values below 90 per cent, whereas the flux through a non-responding membrane with linear response would increase linearly with *a*_in_−*a*_out_ = 0.996 RH. In other words, the SC permeability to water is significantly higher when the skin is exposed to an environment with high RH (greater than 90%) compared with when the surrounding is slightly less humid (RH < 90%). This regulation is likely due to transitions from solid to fluid structures in the SC lipids and corneocyte keratin components upon hydration [30], and it is essential as it regulates the flux of water so that we do not desiccate on a dry day.

In the present study, we are interested in the hydration of the outer layers of the SC, *W*(*z* = 0). We consider the case where we do not alter the intrinsic properties of the SC, and we simply regulate the SC hydration by controlling the boundary condition through a film of cream that is deposited on the outer surface of the skin. If the cream has a high resistance to the diffusive transport of water, the presence of the film will create a new boundary condition for the SC. Indeed, in the steady state, the flux of water across the SC, *J*_w_, must equal the flux of water across the film. This will change the value of *a*_out_, and therefore the value of the hydration in the outer layer of the SC, *W*(*z* = 0). In the following, we show how some properties of the SC will be changed by this new boundary condition, while the regulation of the water flux is maintained.

### 2.2. A new boundary condition for the stratum corneum

We now consider how the conservation of water flux sets a boundary condition at the outer surface of the SC when a film is deposited on the skin. For this purpose, it is sufficient to know the permeability of the SC as a function of the water activity at the skin external boundary, *P*_SC_(*a*_out_), and the permeability of the film, *P*_film_. The steady-state water flux across the SC can be expressed as2.1
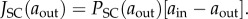


The effective permeability of water in the SC depends on the gradient in water activity in a non-trivial way, and this relation is contained in the function *P*_SC_(*a*_out_). Similarly, the flux across the film is determined by the difference in water activity *a*_out_−*a*_RH_, where *a*_RH_ is the water activity in the atmosphere in contact with air (determined by the RH):2.2
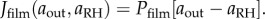


At steady state, the water flux across the skin and the film is equal2.3
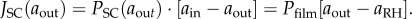


The implication from equation (2.3), is that, knowing the boundary conditions *a*_in_ and *a*_RH_, and the permeabilities of the film and SC under given conditions, it is possible to calculate the water activity at the interface between the skin and the film—that is *a*_out_. For this purpose, we must have a model of the film, as well as a model of the SC. We describe the film as a material with a specific resistance to diffusive transport, *ρ* and thickness *d*_film_:2.4
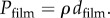


For *P*_SC_, we can use either the predictions from the SC model of responding model membrane [3,29], or experimental results such as those shown in figure 1*a* [7,13,25]. It is here noted that the SC permeability depends on *a*_out_, which must be determined by the conservation of flux. We then solve equation (2.3) numerically, using Newton's method to find a solution to2.5
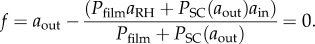


### 2.3. Hydration in the outer layers of stratum corneum

In equations (2.1)–(2.5), we define the permeability as a function of water activity rather than water concentration. Indeed, when treating water flux across a membrane with local inhomogeneities and transport between different media (vapour and liquid, or lipid phase and aqueous phase), it is inconvenient to use water concentration, as this must vary between the different media. The most straightforward way to describe such a system is in terms of the water chemical potential *(Δ*μ**_w_), which is directly related to the water activity (*a*_w_) and the relative humidity (RH) as2.6
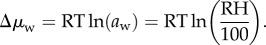


**Figure 2.**
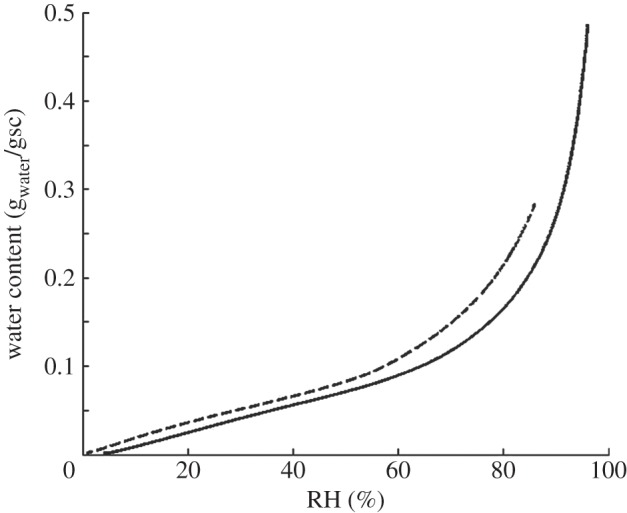
Sorption data for porcine SC [32] for SC from two different animals (solid and dotted line). Water uptake (g_water_/gsc) as a function of RH.

The water uptake in the SC at different RH has been studied experimentally [7,13,31–34]. These studies clearly demonstrate a nonlinear relation between the SC hydration and the boundary condition, *a*_out_. It is a general observation that a large increase in RH leads to a rather minor increase in water uptake in dry surroundings, while a small increase in RH leads to a large increase in water uptake in humid surroundings. The sorption data obtained for SC (figure 2, [31]) can be used to get a relation between water concentration in the upper layer of SC, *W*(*z* = 0), which is in local equilibrium at water activity *a*_out_.

## 3. Novel method to determine the resistance to water transport

The effect of a barrier film on the transport of water is measured by its diffusive resistance *R*, which is the inverse of its permeability *P*. In order to have a measure of this resistance that is only related to the properties of the film and not to its thickness, the specific resistance, *ρ*, is introduced. This is related to the permeability of the film (*P*_film_) and its thickness (*d*_film_) as3.1
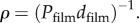


The specific resistance can be obtained from studies of water evaporation across films with varying thickness and at controlled RH. The measurements are performed as measures of water evaporation from an aqueous gel, across the film of interest, to ambient air with known RH. When no film is present on top of aqueous gel, the water flux from the bare water surface (*J*_0_) is determined by the stagnant air layer (*P*_0_) and by the gradient in water activity (*Δa*) between the gel (*a*_gel_ = 1) and the atmosphere (*a*_air_ = RH/100)3.2
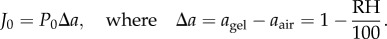


When a film is spread on the aqueous gel and steady state is reached, the water activity at the film–air interface is *a_x_* and the steady-state water flux over the film (*J*) is equal to3.3
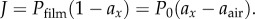


Equation (3.3) can be solved for *a_x_*, and rewritten as3.4
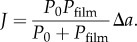


The specific resistance *ρ* can then be determined from the experimental values of *J*_0_ and *J* for any film with known thickness *d*_film_, using equations (3.2)–(3.4)3.5
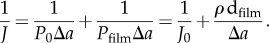


If *J*_0_, *ρ* and *Δa* are constant, then there is a linear relation between *J*^−1^ and *d*_film_, and the specific resistance can be obtained from the slope of this line.

In the limit where *P*_film_ ≪ *P*_0_ (i.e. *J* ≫ *J*_0_), the major resistance to the flux is lying in the film, and the contribution from the permeability of the stagnant layer is negligible. In this limit, equation (3.4) can be simplified to3.6
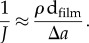


This approximation is also valid when considering water transport across SC as *P*_SC_(*a*_out_) ≪ *P*_0_ for all boundary conditions in water activity.

## 4. Experimental section

The specific resistance was determined for different model emulsions and two examples are given here. The O/W emulsion was prepared with 3 per cent glyceryl monostearate and POE-100 stearate (commercial name Arlacel 165). The oil phase was a mixture of vaseline paste (28%) and beeswax (3%). The emulsion was thickened with 0.3 per cent polyacrylic acid (Carbopol), added to the aqueous phase (water 65.38%) and neutralized by triethanolamine (0.32%). The W/O emulsion was prepared using 1 per cent sorbitan oleate (commercial name Span 80 LQ from Croda). The oil phase was composed of vaseline oil (29%), vaseline paste (28%) and beeswax (1%), and the aqueous phase was water (31%). SVLP (polyvinylidene fluoride) membranes were purchased from Millipore.

### 4.1. Experimental set-up

**Figure 3.**
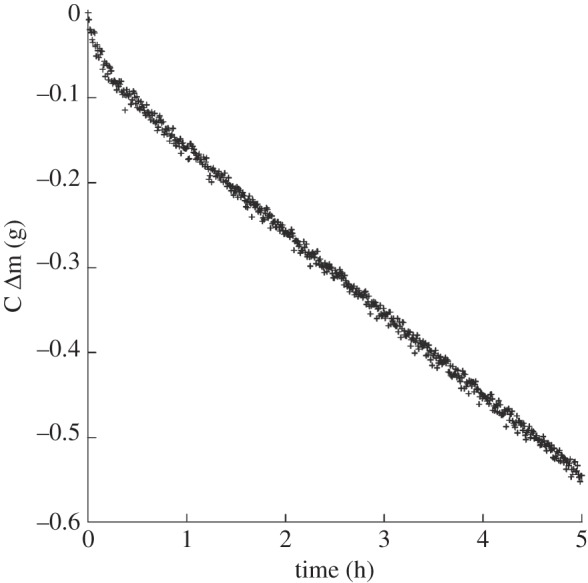
Experimental data for transport of water across a film of the model W/O emulsion (thickness 73 μm, 42%RH).

To measure the specific resistance of different creams, a model system that imitates the water gradient across the skin was used, where the transport of water across the film of cream was studied. The model cream was spread on a SVLP membrane with an automated spreader to a thickness between 50 and 150 µm depending of the viscosity of the cream. The supporting membrane with the film was then applied to the top of a 2 per cent Carbopol gel placed in a 0.082 m diameter container. The weight loss was measured in a RH chamber at 32°C and 40%RH, and recorded as a function of time. One example is shown in figure 3. Initially, the weight loss is related both to drying of the emulsion film and water evaporation from the aqueous gel across the film. When steady state is reached, a linear relation between weight loss and time is obtained, and the constant slope of this line can be used to calculate the water flux across the supporting membrane with the film of cream.4.1
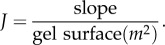

The specific resistance is then obtained according to equations (3.5)–(3.6). It is an important observation that for the barrier films studied, the measured flux is proportional to the area of the container, and no effects of increased flux at the film perimeter could be concluded for these systems. This would not be the case for evaporation from the free surface of an aqueous solution [35].

## 5. Results

### 5.1. Model calculations of permeability and transepidermal water loss for the skin + film composite

**Figure 4.**
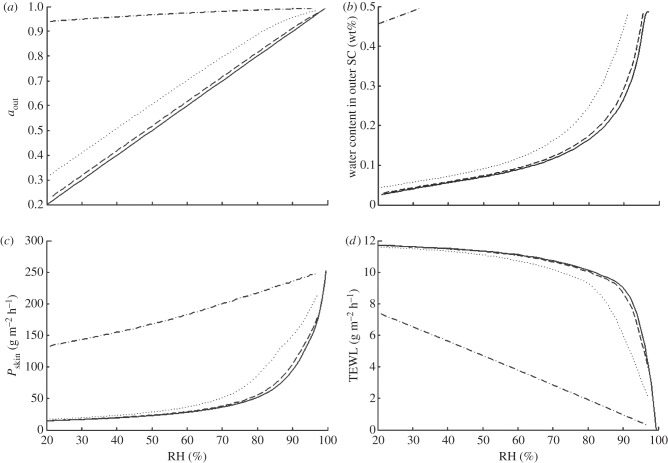
The effect of an occluding barrier cream on the water activity at the skin surface, skin hydration, skin permeability and TEWL. (*a*–*d*) Calculated profiles for skin covered by 20 μm thick films of different specific diffusive resistance (*ρ* = 100 (dashed line), 500 (dotted line) and 5000 (dashed-dotted line) m h^–1^ g^−1^) and for bare skin surface (solid line, no film). (*a*) Water activity *a*_out_ as a function of RH, (*b*) hydration in the very upper layer of SC (wt% water in SC) as a function of RH, (*c*) water permeability of SC as a function of RH and (*d*) water flux across SC (TEWL) as a function of RH.

The theoretical model can be used to calculate the water activity at the skin surface *a*_out_, the TEWL and the permeability *P*_SC_ for SC covered by any barrier film with known *ρ* and *d*_film_. Some example calculations are shown in figure 4. The examples are chosen as representative of the specific resistances measured for different creams. When there is no film, the water activity at the skin surface is the same as that in the surrounding air (*a*_out_ = *a*_RH_). When the film is applied to the skin surface, *a*_out_ increases, and the effect is most prominent at low RH (figure 4*a*). For films with low resistance (*ρ* < 100 m h^−1^ g^−1^), *a*_out_ ≈ *a*_RH_, and the environment of the skin is not different from the RH in the surrounding air. In the presence of a film with extreme resistance (*ρ* = 5000 m h^−1^ g^−1^), the skin environment is quite humid. According to the calculations, it takes a specific resistance of the order of 500 m h^−1^ g^−1^ to produce a significant increase in the water activity at the skin surface, *a*_out_.

A natural consequence of the increase in *a*_out_ is that the skin becomes more hydrated [6]. The relation between water uptake and *a*_out_ has been studied separately. In the present study, we use experimental data for porcine SC hydration at varying RH [31]. Similar data for water uptake have been reported for human, porcine or neonatal rat SC [7,13,32–34]. All these studies were made on intact SC, separated from epidermis, at equilibrium (so there was no gradient, and the water activity was *a*_w_ = RH/100 everywhere). In the steady-state situation, the very upper layer of the SC is in local equilibrium at water activity *a*_out_. Therefore, we can use the equilibrium hydration data at a given activity to estimate the steady-state water content of the very upper layer of SC, *W*(*z* = 0), at the same water activity. This leads to a prediction of changes in hydration in the upper layer of SC as illustrated in figure 4*b* and table 1. When going deeper into the SC, the water activity increases and in the lower part it reaches 0.996 (physiological conditions). This means that the SC hydration varies along the gradient in water activity [9], and the driest layer of SC is the one facing the film (or air in case no film is present).

**Table 1.** Calculated data for the hydration (g/g) of in the upper layer of skin covered with cosmetic films. Film thickness refers to the thickness of the film when steady-state water flux is reached.

RH	no film^a^	20 μm film (*ρ* = 100 m h^−1^ g^−1^)		20 μm film (*ρ* = 500 m h^−1^ g^−1^)	
	SC_surface_ hydration (g/g)	SC_surface_ hydration (g/g)	increased hydration (%)^b^	SC_surface_ hydration (g/g)	increased hydration (%)^b^
25% RH	0.032	0.035	9	0.048	50
50% RH	0.066	0.069	3	0.083	25
75% RH	0.12	0.13	5	0.17	36
90% RH	0.21	0.23	7	0.31	46

^a^Bare skin with no film.

^b^The percentage increase is here calculated as the increase compared with the SC_surface_ hydration for the bare skin at the same RH{*c*_w,film_(RH) − *c*_w,SC_(RH)}/*c*_w,SC_(RH).

Figure 1*a* shows that the water permeability of SC is lower when the water gradient is large (i.e. in dry conditions) compared with situations when the water gradient is small (humid conditions). This implies that the barrier properties of healthy skin are regulated by variations in water activity [7,9,13,15,25]. Since we know the water activity *a*_out_ for skin covered by a barrier film, we can calculate the effect of films with different barrier properties on the permeability of the SC using equations (2.1)–(2.5) (figure 4*c*). For example, the water permeability of SC beneath a 20 μm film with *ρ* = 600 m h^−1^ s^−1^ at 80%RH is twice that of the bare SC at the same RH. Hydration of the SC leads to increased in the mobility in both SC lipid and protein components [30], and most likely these also facilitate molecular diffusion of most small molecules. Thus, the hydration of SC does not only lead to increased permeability of water in SC, but also an increased permeability of many other small molecules in SC [8,27,28]. This implies that the application of a barrier cream may also be used to enhance penetration of active components from the formulation into the skin, which is also used in practical applications in transdermal drug delivery.

Figure 4*d* shows the predicted TEWL for skin covered with different films. The magnitude of TEWL is determined by the total gradient in water activity (*a*_RH_ − *a*_in_), the water permeability of the SC for this boundary condition *a*_out_, and the film resistance to water transport, *ρ*. The curve ‘no film’ shows experimental data from Blank *et al*. [7], and it demonstrates the character of a responding SC membrane. The application of a film with very high *ρ* leads to a significant decrease in water flux as this film presents an efficient barrier to the water flux. However, for films with relatively high *ρ* (e.g. *ρ* = 500 m h^−1^ g^−1^), there is hardly any effect of the film of barrier cream on the TEWL at RH < 80%. Indeed, the calculations show that the application of films with *ρ* < 1000 m h^−1^ g^−1^ has very minor effect on the TEWL. This can be explained by that the resistance to water loss of the whole barrier (SC + film) is the sum of the resistance in SC and the resistance in the film. The result is that the effective permeability remains similar to that of bare skin at the same RH in most cases. In this way, the skin regulates the flux, even when covered with a passive barrier film.

### 5.2. Combining experiments and calculations

We have described a method for experimentally determining the film resistance to water transport, and a theoretical model for a responding skin membrane and a film. In this section, we combine these to predict the effects of films composed of cosmetic or pharmaceutical formulations on water activity at the SC outer surface, on SC hydration, TEWL and skin permeability.

Table 2 summarizes some calculated results for the different model formulations. The application of a 20 μm thick film from a typical O/W emulsion film leads to a minor increase in *a*_out_. This increase leads to a small increase in skin hydration as well as water permeability of the skin. For thicker films (50 μm), there is nevertheless a non-negligible increase in skin hydration. For the W/O emulsion with higher *ρ*, the predicated changes in *a*_out_, skin hydration and water permeability are substantial even for 10 μm thick films. The skin permeability increases with increasing *a*_out_, which is a consequence of that skin is a responding membrane. The effects are most pronounced at low RH.

**Table 2.** Combination of experimental data and model calculations. Water activity at the skin surface, skin hydration, skin permeability and TEWL for skin covered different model emulsions. Film thickness refers to the thickness of the film when steady-state water flux is reached.

model formulation	*ρ*^a^ (m h^−1^ g^−1^)	RH (%)	*d*_film_ (μm)	*a*_out_^b^	*J*_w_^b^ (g h^−1^ m^−2^)	*P*_SC_^b^ (g h^−1^ m^−2^)	increased hydration (%)^b,c^
model O/W emulsion	58	50	10	0.50	11	23	0
			20	0.51	11	23	2
			50	0.53	11	24	6
		25	10	0.25	12	16	0
			20	0.26	12	16	4
			50	0.28	12	16	14
model W/O emulsion	423	50	10	0.54	11	25	9
			20	0.59	11	27	21
			50	0.73	11	40	73
		25	10	0.29	12	17	21
			20	0.35	12	18	43
			50	0.49	11	23	102

^a^Experimental results for the specific resistance to water for some model formulations.

^b^Calculated values of water activity (*a*_out_), TEWL (*J*_w_), skin permeability (*P*_skin_) and the increase in hydration for the very upper layer of skin for skin covered with different model emulsions.

^c^The percentage increase is here calculated as the increase compared with the SC_surface_ hydration for the bare skin at the same RH:{*c*_w,film_(RH) – *c*_w,SC_(RH)}/*c*_w,SC_(RH).

**Figure 5.**
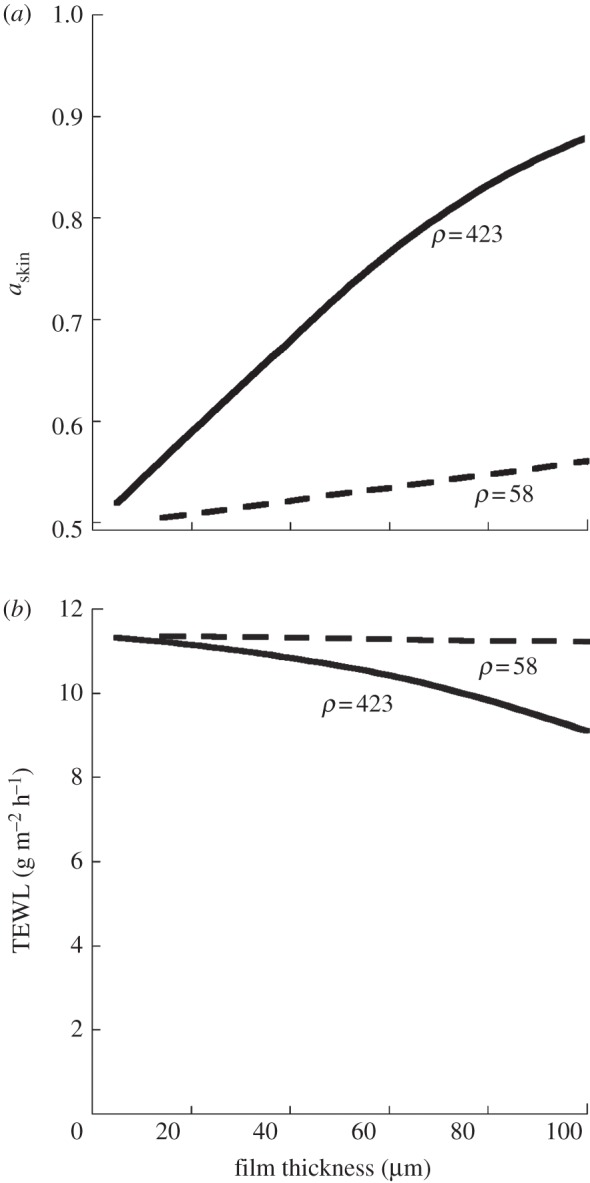
Calculated data for how film thickness influence water activity at the outer surface of the SC and TEWL for the model emulsions investigated (one O/W emulsion with measured *ρ* = 58 m h^−1^ g^−1^, and one W/O emulsion with measured *ρ* = 423 m h^−1^ g^−1^. (*a*) Water activity as a function of film thickness. (*b*) TEWL as a function film thickness.

One notable observation is that the SC regulates the water flux, even when it is covered with a passive barrier film. The explanation for this is that the effective barrier to water transport is determined from the water permeability in SC as well as in the film. Even though *P*_SC_ increases after application of a film, the contribution from the water permeability in the film, *P*_film_ counterbalances this, and the effective permeability remains the same as for the bare SC. In this way, the SC regulates the water flux, even when it is covered with a passive barrier film. Figure 5 illustrates how film thickness affects *a*_out_ and TEWL for the O/W and W/O emulsions at 50%RH. It is shown that *a*_out_ gradually increases with film thickness for both types of films until it approaches 1 for very thick films. On the other hand, no effect on TEWL is seen for the O/W emulsion even with the 100 μm thick film. Even for the highly resistant W/O emulsion film, very thick films (greater than 50 μm) are needed to get any observable effect on TEWL. Together this signifies that TEWL measures are not sensitive to changes in skin hydration for skin covered with barrier films.

## 6. Discussion

In the previous sections, we made quantitative predictions for the effects of barrier creams on the permeability and the hydration of the SC. These predictions are

— In order to have significant effects on the activity of water at the surface of the SC, the resistance to water flux though the barrier film should be in the order of 0.01 m^2^ h^−1^ g^−1^. For a film of thickness 20 µm, this requires a specific resistance of 500 m h^−1^ g^−1^. For SC covered with such film and exposed to the atmosphere at RH = 50%, the activity at the outer surface of the SC increases from *a*_out_ = 0.5 to *a*_out_ = 0.6.
— The effect on the hydration of the outer layer of SC can then be calculated. For the situation described in (a), the hydration in the upper layer of SC would be increased by a factor of 1.26. This increase is significant with respect to cosmetic applications.
— The permeability of the SC increases according to the increase in water activity. For SC covered with the same film as in (a), the SC permeability would increase from 23 to 28 g m^−2^ h^−1^. Yet, for the TEWL, this effect would nearly compensate the additional resistance due to the barrier film. For the film described in (a), the flux would be decreased by 2 per cent only. This is because the SC regulates its TEWL.

To our knowledge, this is the first time that such quantitative predictions can be made. Now we need to examine how reliable these predictions are, what is their range of validity, and whether we could go beyond these predictions?

### 6.1. How reliable are these predictions and what is their range of validity?

The calculations presented in this paper are based on a simple series resistance model for the SC membrane covered with a barrier film. One can say that the SC is treated as a ‘black box’, characterized by a hydration and a permeability that vary according to the boundary conditions in water activity. In this respect, the model is as reliable as the data that are used to describe the response functions of the SC. We used data from Blank *et al*. [7] for SC permeability and data from Silva *et al*. for SC hydration [31]. These data are representative of healthy skin, but of course there are large differences between skin from different parts of the body, and between skin from different individuals. Such variations can easily be incorporated into the model, and the predictions will be as reliable as the input data. The description incorporates its measured response to changes in water activity (i.e. the dependence of permeability *P*_SC_ on water activity), and it shows that this response essential to how the skin barrier works, either alone or in combination with a film applied to it.

The reliability of the predictions also depends on the accuracy of the experimental characterization of the film. A critical point is the quality of the spreading (uniform thickness and the absence of defects in the film). With the instruments described in §3, and for film thicknesses of the order of 100–150 µm, we find that the accuracy on the measurement of film resistance is ±20%. This is much less than the variability in SC resistance. Another critical point is the behaviour of the support membrane, i.e. whether or not the cream will penetrate into the pores of the support membrane. In order to check this, one may remove the film at the end of the experiment and measure the water flux through the membrane alone. This correction for membrane resistance is significant in the case of thin films (20 µm or less). However, the specific resistance of the films is independent of their thickness (as long as the thickness is known), and therefore the measurements can be performed for thick films (100 µm), where this correction is less than the variability from other causes.

The results presented illustrate the effect of a barrier film on normal healthy skin that responds to changes in its environment. Very different results would be obtained for skin that does not regulate its TEWL as healthy human skin does. It is common observations that the TEWL is higher for dry or damaged skin compared with normal skin under ambient conditions [36–38]. In cases where the damaged SC is not able to regulate TEWL (compare non-responding membranes in figure 1*a*), one can predict a stronger effect of the barrier film compared with its effect on healthy skin. The application of a barrier film on the skin surface would then lead to clearly reduced TEWL and to increased water activity at the skin surface. Assuming that the permeability of ‘dry skin’ is *P*_dry_ = 100 g m^−2^ h^−1^ and independent of water activity, this gives a TEWL of 50 g m^−2^ h^−1^ at 50%RH. In this case, we predict that the film will reduce the TEWL considerably, and the same 20 µm thick film of specific resistance *ρ* = 500 m h^−1^ g^−1^ would reduce the TEWL to 25 g m^−2^ h^−1^.

This paper treats the effects of a single application of occlusive barrier creams. Calculated examples are shown for 20 μm thick films, which correspond to the dosage of 2 mg cm^−2^, which is the common recommendation for barrier creams, sunscreens and therapeutic creams [39,40]. The effect of repeated applications of smaller doses is another interesting question, but much more complex since it involves consumer behaviour, and physiology of the skin, and these aspect are not treated here. The comparisons between films with different thickness shown in figure 5 and table 2 demonstrate that the more occluding films can have significant effects on the water activity at the skin surface also for thinner films.

### 6.2. Can we go (do we need to go) beyond the ‘black box’ model of the stratum corneum?

In this work, we restricted our predictions to the outermost layer of the SC, which is at a known water activity, *a*_out_, and we based these predictions on the assumption that this outmost layer of SC is in local equilibrium at *a*_out_. A more detailed description of the responding SC membrane would take into account how the hydration and permeability vary with depth along the gradient in water activity. Indeed, the outer layer of the SC has the lowest water activity of the SC, and it is more dry than the rest of the SC [41]. Nevertheless, a model that includes variations in hydration and permeability with depth would still be constrained to have the same overall permeability as observed experimentally, and for a given value of *a*_out_, it would give the same values of *P*_SC_ and *J*_SC_. The conservation of the water flux, as expressed in equation (2.3), would still hold with the same boundary conditions; and therefore, the value of *a*_out_ would be the same as calculated above. Thus, a gradient model is unnecessary for predicting the boundary conditions of the SC.

Yet, a model that describes the gradient in water activity within SC would provide information on the hydration of the successive layers of the SC, and also provide an estimate of the overall SC hydration. For such a description, it is important to realize that the gradient in water activity varies in a nonlinear way with respect to the depth in SC. At low water activities, the SC components are mainly in a solid state, while hydration of SC leads to increased fluidity in both lipid and protein SC components [30]. The steady-state water gradient is determined from the conservation of flux in each layer within the membrane. From this follows that there will be a large gradient in water activity over a thin solid layer with low permeability in the upper part of SC, and a less steep gradient in water activity over a thicker layer with higher permeability deeper down in SC. These calculations have been previously presented in detail for a responding membrane composed of stacked lipid bilayers as a model for the extracellular SC lipids [3,18,29]. The model requires quantitative data on water diffusion coefficient and water uptake at different water activities. For lipid systems that are well characterized in these respects, we were able to make the quantitative prediction that less than 5 per cent of the bilayers in the upper part of the stack are completely solid [29]. It is an important conclusion that the presence of this thin solid layer strongly reduces the effective permeability of the complete membrane, and this can explain the responding behaviour of SC as illustrated in figure 1*a*. It should also be pointed out that the water permeability is mainly determined by the properties of the non-aqueous domains in the membrane (lipids and proteins), which are affected by changes in the water activity. However, the variations in the water concentration as such have no significant effect on the water flux, as the aqueous domains do not significantly contribute to the resistance of the flux [3].

Once the profile in water activity is known, it is possible to estimate the variation in water content at different positions in the membrane, assuming local equilibrium with the local water activity at each position [29]. This requires a relation between water activity and water content, which can be derived from experimental data (e.g. water sorption isotherms), or from theoretical analysis of, e.g. interlamellar interactions. Here, the ability of the system to swell in water is determined from its properties in terms of charge, phase state, etc. [42]. The calculations for the responding lipid model membrane in figure 1 were performed for a lipid bilayer systems composed of uncharged and anionic lipids [3,29]. Similar behaviour is expected for charged keratin rods [43,44]. It is clear that the model for the membrane composed of stacked lipid bilayers captures the important features in the data obtained for intact SC both with respect to permeability and hydration.

In summary, a model that describes the water gradient within the SC is not necessary for predicting the boundary conditions of the SC in a skin/film composite. However, such model can help to give a more precise definition of the extension of the solid outer layer in SC that controls the permeability. Moreover, it can demonstrate what physical phenomena give the nonlinear response of the SC to changes on water activity. A gradient model for SC would require additional experimental data for SC (e.g. water diffusion coefficient at varying water activities) or simplifying assumptions, for example, treating SC as a bilayer stack as previously described [3,18,29].

## 7. Conclusions

One of the most important properties of a cosmetic cream is its barrier property when spread as film on the skin surface. By applying on the skin surface, a cosmetic film with a high-specific resistance to water transport, it is possible to alter the resistance to water evaporation from the body and to increase the water activity at the skin surface. This way, the spreading of a barrier film is an effective and non-invasive way to increase skin hydration.

We have constructed a theoretical model to calculate the influence of films with high-specific resistance to water transport on skin hydration and skin barrier properties. In particular, we study the effect of the film on the water activity at the interface between the skin and the film. The model treats water transport across a barrier that consists of a responding skin membrane and a film. We show that the diffusive resistance of the film determines water activity at the upper surface of the SC. A change in water activity can affect the skin hydration, and we predict this change of hydration in the upper layer of the SC for films with different thickness and specific resistance.

We also presented an experimental method to determine the specific resistance of films. From the combined calculations and experiments, we are able to make quantitative predictions for how films of different formulations affect the water activity at the skin surface, TEWL and the hydration of the upper layer of SC. For most cosmetic O/W emulsions investigated, the specific resistance was rather low, while higher values for the resistance were observed for W/O emulsions. Even though the increase in skin water activity caused by a film of an emulsion with low resistance is limited, it can lead to a significant increase in skin hydration. This effect is most pronounced at low RH and for thicker films. The skin permeability also increases and this increase compensates the added resistance due to the film, so that the TEWL is nearly unchanged. This compensation is a consequence of that skin is a responding membrane.
